# Experiences with the levonorgestrel-releasing intrauterine system in Kenya: qualitative interviews with users and their partners

**DOI:** 10.1080/13625187.2018.1499892

**Published:** 2018-09-10

**Authors:** Geeta Nanda, Kate Rademacher, Marsden Solomon, Sarah Mercer, Jim Wawire, Rose Ngahu

**Affiliations:** a Family Health International (FHI 360), Washington, DC, USA;; b Family Health International (FHI 360), Durham, NC, USA;; c Family Health International (FHI 360), Nairobi, Kenya;; d Austin, TX, USA, formerly with Family Health International (FHI 360), Durham, NC, USA;; e Family Health Options Kenya (FHOK), Nairobi, Kenya

**Keywords:** Kenya, LARC, levonorgestrel-releasing intrauterine system, LNG-IUS, long-acting reversible contraception, Mirena, partner perspective

## Abstract

**Objectives:** The levonorgestrel-releasing intrauterine system (LNG-IUS) is an underused contraceptive method in sub-Saharan Africa. A recent market assessment in Kenya found that if a more affordable version of the method were available it may increase demand and uptake of the method. We therefore aimed to examine attitudes and perceptions around the LNG-IUS and experiences of method use, including exploring attributes such as bleeding changes, contraceptive-related amenorrhoea and perceived non-contraceptive benefits.

**Methods:** Qualitative interviews were conducted among 29 women who were current or recent users of the LNG-IUS, and among a subset (*n* = 9) of their husbands/partners.

**Results:** Our findings indicate that women’s main reason for choosing the LNG-IUS for contraception was their perception that the method had fewer side effects compared with other contraceptive methods. Women had favourable attitudes towards using the LNG-IUS. Husbands were also very positive about their partner’s use of the method.

**Conclusion:** Understanding the motivations and experiences of early adopters of the LNG-IUS can help inform the development of demand creation and communication strategies to influence uptake and continuation of the LNG-IUS both in Kenya and perhaps more broadly. Communication efforts that emphasise the positive attributes of the LNG-IUS could help promote wider use of the method, especially if new, more affordable product(s) become available.

## Introduction

In low-income countries, around 214 million women wish to delay or avoid pregnancy but are not using a modern contraceptive method, and an estimated 89 million unintended pregnancies occur annually [1]. While some women do not have access to effective contraceptive methods, owing to social, cultural or economic factors, other reasons for non-use or discontinuation include, for example, side effects or health risks perceived or experienced with method use [2–4]. Short-acting hormonal methods, such as oral contraceptive pills and injectables, are beset by low adherence and/or low continuation rates due to the need for frequent follow-up visits for resupply [5]. Use of the intrauterine device (IUD), specifically the copper T 380A IUD, which is a long-acting reversible contraceptive (LARC) method, is currently the most widely used reversible contraceptive method globally [6]. However, the geographic distribution of copper IUD users is not uniform since most users (83%) live in Asia [7]. In sub-Saharan Africa, copper IUDs are used by only 2% of women. The hormone-containing intrauterine system, known as the levonorgestrel-releasing intrauterine system (LNG-IUS) (Mirena; Bayer HealthCare Pharmaceuticals, Berlin, Germany), another LARC method, is one of the most effective forms of reversible contraception available, with a 5-year failure rate of <1% [8]. Features of the LNG-IUS that could be of significant benefit to women in low-resource settings include its long-acting duration, reduced menstrual cramps and bleeding, fewer side effects relative to other hormonal methods, and potential alleviation of anaemia. Although the LNG-IUS is increasingly popular among women where it is available, particularly in U.S.A. and Europe, its high cost has limited its availability and use in low- and middle-income countries [9–11].

In Kenya, in 2016, the modern contraceptive prevalence rate was 59.9% [12]. The dominant modern method used is injectables. Implant use, however, has increased from 1.9% in 2008–2009 to 9.9% in 2014, and up to 30.2% in 2016 [12–14]. By contrast, copper IUD use remains relatively low at 5.9% [12]. The LNG-IUS is not currently reflected in national surveys because of its low prevalence. The 5-year LNG-IUS is only offered on a limited basis in the private sector in Kenya and at a relatively high price; typically, only high-income women have been able to access the method to date [9,15]. Despite low availability of the method, recent evidence suggests that the LNG-IUS could fill an important role in the method mix in Kenya if it were more widely available [16,17].

A global not-for-profit pharmaceutical company, Medicines360, is poised to introduce a new, highly effective LNG-IUS product that will be priced to ensure more affordable access in low-income countries, including Kenya. In 2016, findings from a market assessment suggested that introduction of a new, more affordable LNG-IUS could drive increased awareness, demand and uptake of the method [9]. Building on the earlier market assessment, the current project included interviews with current or recent LNG-IUS users in Kenya and a subset of their male partners, to further understand women’s and men’s needs and preferences. Our primary objective was to examine attitudes and perspectives regarding product-related acceptability and attributes such as bleeding changes, contraceptive-related amenorrhoea and perceived non-contraceptive benefits. To our knowledge, literature on previous qualitative research from sub-Saharan Africa on users’ experiences with the LNG-IUS is very limited [18]; this article is an attempt to address the evidence gap.

## Methods

In June–July 2016, we conducted 38 qualitative interviews in two Family Health Options Kenya (FHOK) clinics in Nairobi, including 29 with current or recent LNG-IUS users and nine with a subset of their male partners. We chose these facilities based on their relatively high-volume distribution of the LNG-IUS in recent years. The current project was carried out in partnership with a local non-governmental family planning organisation in the private sector.

Participants were recruited via a convenience sample. We anticipated that interviewing 25–30 women (primary audience) and 10 husbands/partners (secondary audience) would be adequate to address our objectives. Using clinic records, two service providers, one at each selected family planning clinic, made the initial contact with potential participants by telephone to introduce the project and screen for eligibility. Participants were eligible if they were LNG-IUS users who had used the method for at least 6 months and were either still using it or had recently discontinued, were between 18 and 49 years of age and were willing to give informed consent. We asked female participants to help us recruit their husbands/partners for an interview as well, if they were between 18 and 49 years and willing to give informed consent. We trained data collectors to conduct the qualitative interviews in person or by phone for participants who did not have the time to meet in person. For interviews conducted in person, participants were reimbursed 800 KSH (USD 8) for their travel expenses.

The qualitative interviews were conducted in either English or Swahili, depending on the client’s preference, using a semi-structured guide. The interviews were audiotaped and transcribed verbatim, with translation included simultaneously for interviews conducted in Swahili. The team reviewed transcripts and conducted a thematic content analysis. Two analysts created codes based on themes of interest. One analyst systematically moved through the transcripts coding words, phrases and statements into the appropriate coding category. The analysis included the coding as well as identification of patterns of topics and themes not previously identified. The data were grouped and regrouped, focused, sharpened and organised so that the final conclusions were distilled from participants’ responses. A qualitative software package (NVivo, version 11.0; www.qsrinternational.com/nvivo/home) was used to help analyse the data.

Both FHI 360’s office of international research ethics and a local institutional review board in Kenya approved the project. All participants voluntarily enrolled through the approved informed consent process.

## Results

Participants’ demographic information is shown in Table 1. We interviewed 29 female and nine male participants. Owing to difficulty in arranging a convenient time for participants to meet in person with interviewers, seven participants were interviewed by phone. The mean age of all participants was 38 years. Most were married, had approximately two children, and were educated professionals in technical or managerial positions. Among women, 23 were currently using the LNG-IUS and six had recently discontinued use. Women cited a range of reasons for discontinuing: two women had recurring infections; one woman wanted to get pregnant; one woman no longer needed the method as her husband had passed away; and two women reported that that they did not like the changes to their periods, specifically spotting and not having regular periods. Previous contraceptive method use was most commonly oral contraceptives followed by the copper IUD and injectables. More than half reported no desire for children in the future. Women reported paying from 4000 to 20,000 KSH (USD 40–200) for the LNG-IUS and its insertion, with an average price of 11,138 KSH (USD 111).

**Table 1.** Participants’ sociodemographic characteristics and family planning (FP) use.

Characteristics and FP use	Women (*n* = 29)	Men (*n* = 9)	Total (*N* = 38)
Age in years, *n*, mean	38.3	38.7	38.4
Marital status, *n*			
Single	1	0	1
Married	26	9	35
In union/living together	1	0	1
Widowed	1	0	1
No. of children, *n*, mean	2.3	2	2.2
Education, *n*			
Completed primary	1	0	1
Some secondary	1	0	1
Completed secondary	3	0	3
Post-secondary	24	9	33
Occupation, *n*			
Professional/technical/managerial	22	8	30
Other	7	1	8
Currently using LNG-IUS, *n*	23	6	29
Recently removed LNG-IUS, *n*	6	3	9
FP methods ever used, *n*^a^			
Condoms	1	5	6
Pills	17	4	21
Injectables	7	4	11
Implants	6	3	9
Copper IUD	8	3	11
Other^b^	4	5	9
Desire for future children, *n*			
Yes	8	6	14
No	18	3	21
Maybe	3	0	3
Average amount paid for LNG-IUS, KSH (USD)	11,138 (111)	–	–

Only one woman reported having no children.

a Not mutually exclusive.

b Includes herbs, natural method, counting days and withdrawal.

### Women’s reasons for choosing the LNG-IUS

Interviews revealed a range of reasons why women chose the LNG-IUS; most common was their perception that the method had fewer side effects compared with other methods, particularly because it was understood to contain lower hormone levels. Specific side effects that women wanted to avoid included weight gain and ‘hormonal imbalances’. A first-time user of the method who had previously tried other methods that caused her weight to fluctuate explained that she liked the lack of side effects as well as the convenience:

The fact that it didn’t give me any imbalances or such weight gain or something … [I didn’t] actually experience any of that. And the convenience; once it is put you are done. (age 32 years, two children)

Another participant who weighed perceived risks associated with use in her decision making chose the LNG-IUS because she ‘*didn’t want anything that would mess up my hormones*’. She researched information on the internet related to the LNG-IUS, including women’s testimonials about positive use, and said the information she read made her feel more comfortable about using the method:

Even though hormones are involved, it is at a very minimal level; something that doesn’t put my life at a risk … the amount of hormone is very little and it is not something that gets into my bloodstream, so I was very happy with that. (age 37 years, three children)

Women knew the LNG-IUS contained hormones, but also understood that because of the local release of the hormone within the uterus the LNG-IUS has a lower systemic dose of hormones compared with other methods. As one participant who selected the LNG-IUS for spacing explained:

Just tell them to go for it; it works well. It works well, [laughs] because most women, you know, fear adding weight. You can maintain your shape by doing the right thing, taking the right food and exercising. There is no need for you to worry that you will add weight, because the hormone is just localised along the cervix; it does not go to the bloodstream. (age 35 years, three children)

One woman who had previously used pills wanted to switch to another method due to the side effects she reported with the pill, including headaches and weight gain. She learned about the LNG-IUS from her health care provider during her clinic visit when seeking another method:

I didn’t want something which would interfere with, you know, the whole body system, like hormones … interfere with the whole system. So, the way I understood it, I thought it would affect only … you know that part which is concerned. So that is why I went for it. Now that I thought it will not interfere with me … it will not work like the other hormones. (age 42 years, two children)

Other important reasons for choosing the LNG-IUS were duration of effectiveness, perceived non-contraceptive health benefits such as treatment for fibroids, and recommendations from friends and health care providers. Participants considered the long-acting duration of the LNG-IUS method a reassuring and low-maintenance feature that did not require them to take action daily or return to the provider on a regular basis. As one participant explained:

It is something I don’t have to pop into the mouth every day. And it is something that keeps me going for about 5 years, then that made all the sense to me. (age 37 years, three children)

One woman who had been a copper IUD user but was advised by a nurse to try the LNG-IUS for treatment of her fibroids said:

Mainly I wanted to reduce the fibroids that were growing. When I heard the Mirena can stop the growth of fibroids that is the time that I said OK. (age 48 years, three children)

Another participant had been an implant user but was not happy with the incisions in her skin and felt the removal process of that method was not easy. She heard about the LNG-IUS from her friends and understood that it would provide similar benefits to those of the implant:

So other friends referred Mirena in terms of it is hormonal, so chances of conception are reduced … and you don’t bleed much. That is what used to make me shy away from the IUD is the heavy bleeding, but apparently the people who have used it [the LNG-IUS] say bleeding is minimal … so I got the convenience of what I am using … I considered the effectiveness … the benefits of implant I will still get from now the IUD … without now having to insert it in my skin. (age 41 years, two children)

Along similar lines, a health care provider was instrumental in the following participants’ decision to use the LNG-IUS:

I think I trusted the doctor. The manner in which he spoke to me … I would say that he had good communication skills because somehow he made me have no doubt that this is one good product. That is how he, you know he really brought out the goodness of the product when he was talking to me … I think he may have really persuaded me to use it and he was not wrong on this. (age 42 years, two children)

### Women’s general perceptions of using the LNG-IUS

After they were asked about their reasons for choosing the LNG-IUS, participants were asked about their experiences with using the method. Almost all women reported a positive experience, the most frequently mentioned attributes being convenience of the method/not having to worry about resupply, not experiencing side effects, the longevity of the method, and having reduced periods:

It is 5 years. For all these 5 years I don’t have to worry about pregnancy. I have not heard anybody say it has failed. It empowers a woman. (age 36 years, two children)

Mirena is clean … there are no side effects … I don’t feel any pain, I don’t feel nausea, and I feel like a twelve year old … I am full of energy, full of vibe and full of life. (age 41 years, one child)

Another participant said:

For me, the major thing it is comfortable … and I don’t get my periods [laughs]. (age 35 years, two children)

This woman, who was currently on her third LNG-IUS, had initially gone to her health care provider to seek reassurance about no longer having monthly bleeding and was told by the provider that this was normal with use of the method.

Most women did not have anything negative to say about the method, as expressed here:

You know if you have never experienced anything negative you cannot say anything. (age 46 years, two children)

Among those who did mention negative aspects, however, these were most often related to the discomfort of the insertion procedure and the high cost of the method:

Maybe the cost: it is on the higher side, especially if you are used to buying pills over the counter for 30 shillings … and here you have to pay 10,000. (age 36 years, two children)

The insertion was not comfortable at all, at all … it was quite uncomfortable. (age 36 years, three children)

Given the widely expressed positive perceptions around use of the LNG-IUS, most women said they would recommend it to their friends, citing factors discussed above such as their perception that the method lacked side effects, was convenient and was effective at preventing pregnancy:

I talk of the minimal … almost zero side effects it has. I talk about the freedom it gives a woman in terms of menses. (age 36 years, two children)

### Menstrual bleeding changes with LNG-IUS use

We asked women specifically about menstrual bleeding changes, as they are a common side effect reported with LNG-IUS use. A small number of women reported heavier periods (three women), or irregular periods (three women), with LNG-IUS use, and they did not like these changes; two of these women ultimately discontinued use of the method and one was considering discontinuing the method if her heavy bleeding did not stop. Another woman reported that her periods completely stopped after using the LNG-IUS. Most women reported either that their periods remained the same or that they had reduced periods, including light periods or just spotting; and they liked these changes:

I don’t feel it. My periods are reduced, almost completely gone; like I would say, I actually just spot … so for me it is very convenient … I mean I am basically not conscious that I have my period. (age 41 years, two children)

The following participant who was advised to try the LNG-IUS for treatment of her fibroids said she also liked the resulting changes in her period:

The bleeding days, it is not heavy and my days are shorter actually … when you have Mirena you are free, your bleeding is not heavy and you feel free. (age 44 years, four children)

In some instances, women indicated that although their periods were currently normal or lighter, they had initially experienced heavier bleeding when they had first started using the LNG-IUS. One woman turned to an online social media group to seek reassurance about the changes that were taking place with her period:

The first 3 months I almost removed it. I bled and … I bled. But … thanks to social media, I asked … and I was told it was normal so I held on for 3 months, the bleeding stopped and now it is pretty comfortable … it is not exactly regular but it is very light … I am happy with it. (age 36 years, two children)

### Perspectives of male partners on the LNG-IUS

Male participants were asked about a variety of topics like those asked of female participants, including their views on why their partner chose the LNG-IUS and their experiences using the method. Men reported a range of reasons for why their partner chose the LNG-IUS, including effectiveness in preventing pregnancy, having no side effects, flexibility in terms of when one can remove or stop using the method, safety, and cost-effectiveness of the method over time. For example, as stated by one husband:

We gave it a try because she was tired of the [side] effects of those others. (age 38 years, two children)

Two men reported that their partners chose the method for treatment of heavy bleeding or fibroids, as the LNG-IUS had been recommended by their health care provider.

Most men reported being satisfied with their partners’ and their own experiences in using the LNG-IUS. Some of the reasons men cited were that the method had been effective in preventing pregnancy and that their partner had not experienced any side effects, including that libido was not affected. For example, as explained by one man:

She does not complain about it like the way she was talking about pills that could make her legs a bit swollen … and she could have emotional imbalance. This was with the pills; but with this one she says it is good and it does not affect her. She does not remember she has it. (age 35 years, two children)

Another participant said he was generally satisfied with the method but could feel the string of the LNG-IUS during intercourse and that it was uncomfortable for him:

It feels solid like you feel the wires, so it’s uncomfortable. (age 35 years, two children)

Two men said they were not satisfied with their partner’s use of the method because of side effects: one woman was having irregular periods and weight gain, and the other woman complained of swelling and painful breasts. One of these participants indicated that his wife had already had the device removed at the time of the interview; the other mentioned his partner would have it removed if the side effects did not improve.

## Discussion

### Findings and interpretation, and comparison with other studies

We found that women’s main reason for choosing the LNG-IUS was their perception that the method had fewer side effects compared with other methods. The 5-year LNG-IUS releases approximately 20 μg of the hormone levonorgestrel directly into the uterus each day. The mechanism of action is primarily local; systemic blood levels of the hormone are substantially lower with the LNG-IUS than with other hormonal methods such as implants and oral contraceptive pills [19,20]. It is notable that participants understood this and found this attribute attractive when they were deciding which method to use. This is a particularly important finding given that the most common reason that women in developing countries cite for non-use of contraception is concern about side effects and health risks [4]. Moreover, an important reason why women may discontinue using a contraceptive method (other than because they want to get pregnant or no longer need pregnancy prevention) is because of ‘method-related concerns’, including side effects, whether experienced or perceived [21]. As such, the LNG-IUS may be attractive to women who seek a method where, as one women interviewed said, ‘*hormones are involved … at a very minimal level.*’

Having a friend or a health care provider who recommended the method was a common reason mentioned for selecting the LNG-IUS. This finding is consistent with previous research that identified providers and friends as trusted sources of information on family planning [22,23]. Following adoption of the LNG-IUS, almost all participants currently using it reported positive experiences. Most men who were interviewed were also very positive about their partner’s use of the LNG-IUS. Recent research in Kenya revealed that high quality of counselling by providers—including support in selecting a method and provision of information on side effects—was positively associated with modern contraceptive use [24]. Evidence also suggests that women trust members of their social network for information about contraception [25,26]. Likewise, women’s contraceptive decision making is influenced by support, or lack thereof, from male partners [27].

The positive attributes that women mentioned were the convenience of the method and not having to worry about getting pregnant, not experiencing side effects, the duration of the method, and having reduced periods. Notably, the perception that the LNG-IUS had fewer side effects compared with other family planning methods was both a key reason in choosing the method and an important aspect of what women liked about the method once they began using it. These findings are consistent with those from a qualitative study of LNG-IUS users in Ghana in which women frequently cited similar method benefits such as long-term protection and reduced menstrual bleeding [18]. Similarly, recent qualitative interviews with LNG-IUS users in Nigeria found that the most common reason reported for choosing the method was because of a health care provider’s recommendation (work in preparation by first author and others). Moreover, when asked about perceived advantages, LNG-IUS users in Nigeria most often mentioned that they liked not having any side effects with the method compared with other methods.

Most women reported either that their periods remained the same or that they had markedly reduced menstrual bleeding, and they generally liked these changes. These findings are consistent with those from a previous cohort study of LNG-IUS users in Kenya; among those who reported having menstrual bleeding changes, the dominant pattern of which was spotting, 75% felt their bleeding pattern was highly acceptable [17]. Women we interviewed also reported that they appreciated the reduced bleeding associated with LNG-IUS use for its non-contraceptive health benefits (e.g., as treatment for menorrhagia and fibroids), as well as lifestyle advantages (e.g., increased convenience and freedom). This is an important finding, as limited research has been conducted in developing countries about women’s attitudes towards contraceptive-induced bleeding changes, and there is often a perception that women in Africa will not find reduced bleeding acceptable [9].

### Limitations and strengths

A key limitation of this study is that the participants were highly educated professionals and thus probably of a higher socioeconomic status that is not typical of the Kenyan population. As a result, these findings may not be applicable or generalisable to other settings or to Kenyan women of low socioeconomic status. Nonetheless, we gained valuable insights into the motivations and experiences among these ‘early adopters’ of the LNG-IUS. Early adopters can often act as change agents who have the potential influence to help shape contraceptive markets [28]. In addition, results from this project may be used by service delivery partners to help inform the development of demand creation and communication strategies for influencing uptake and continuation of the LNG-IUS both in Kenya and perhaps more broadly. As noted above, participants were keen to avoid contraceptive-related side effects; their perception that the LNG-IUS had fewer side effects compared with other modern methods was an important factor in its selection. This is particularly relevant in the Kenyan context since the proportion of currently married women citing ‘health concerns’ or ‘fear of side effects’ as the main reason for contraceptive non-use increased from 15.6% in the 1993 Demographic and Health Survey (www.statcompiler.com), to 22% in 2003 and 29.5% in 2014 [13,14].

## Conclusion

As the international community and both national and local programmes seek opportunities to reduce the global unmet need for family planning, the LNG-IUS could play an increasingly important role in the method mix. In addition to being a highly effective LARC method, the LNG-IUS could be positioned as a method with fewer hormones—and potentially fewer side effects—compared with other contraceptive methods. The menstrual bleeding profile could also be promoted as an advantage for both health and lifestyle reasons. Findings suggest that women would benefit from counselling efforts that manage expectations around bleeding and provide reassurance about the safety of reduced bleeding and amenorrhoea [17]. Communication efforts that emphasise the positive attributes of the LNG-IUS could help promote wider use of the method, particularly if new, more affordable product(s) become available.
